# Metabolic Parameters in Asian Patients Under the Early Intervention Service in Sheffield

**DOI:** 10.1192/bjo.2026.11220

**Published:** 2026-06-30

**Authors:** Eleanor Beard, Hamna Tehniyyat

**Affiliations:** Sheffield Health Partnership University, Sheffield, United Kingdom

## Abstract

**Aims:**

There is a high prevalence of metabolic abnormalities in patients with severe mental illness. Metabolic abnormalities are more likely to be present among individuals of black, Asian or minority ethnic groups. This study aims to assess the ethnic breakdown of the Early Intervention Service (EIS) for first episode of psychosis in Sheffield Health Partnership University NHS Trust. We aim to assess the metabolic parameters (blood pressure, HbA1c, BMI, triglycerides and cholesterol) in Asian patients on the EIS caseload.

**Methods:**

Data on ethnicity was collected for all patients on the EIS caseload in 2025 using electronic patient records. Data on BMI, blood pressure and blood results were collected for patients of Asian ethnicity. Data was analysed using descriptive statistics and presented on graphs using Microsoft Excel.

**Results:**

Out of the 309 patients on the EIS caseload, 9.2% (29 patients) identified as Asian or Asian British.72% of Asian patients had a recorded BMI. Of these, the majority (71%) had a raised BMI. 13% of patients with recorded data had raised blood pressure. 83% of Asian patients had blood tests for lipids, cholesterol and HbA1c. The mean HbA1c was 36.08mmol/mol and no patients had a HbA1c in the pre-diabetic or diabetic range. 29.2% of Asian patients had a raised TG:HDL ratio.

We used the diagnostic criteria of metabolic syndrome as at least 3 of: waist circumference >102cm for men or 89cm for women, triglyceride >1.7mmol/L, HDL <1.0mmol/L for men or <1.3mmol/L for women, BP >130/85 or fasting BM >5.6. This identified 20.7% of Asian patients as having metabolic syndrome. However, only 3 patients (10.3%) had all 5 parameters recorded, suggesting there could be unidentified patients with metabolic syndrome.

**Conclusion:**

The majority of Asian patients on the EIS caseload had a raised BMI, highlighting a potential area of intervention to improve health outcomes. Some Asian patients have raised blood pressure, increased TG:HDL ratio and metabolic syndrome. This study identifies low rates of measurement of metabolic parameters; it is striking that 89.7% of patients did not have all the recorded data that is needed to make a diagnosis of metabolic syndrome. It is likely that there are unidentified patients with metabolic syndrome who are not able to access appropriate lifestyle support and medical treatment to improve health outcomes.

